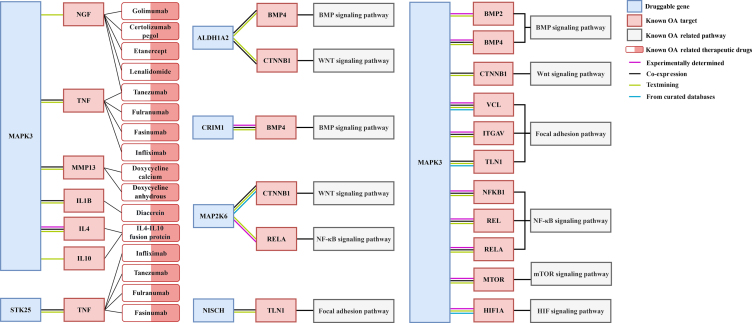# Multi-omics Mendelian randomization combined with single-cell and spatial transcriptomics: Multidimensional validation of drug targets for osteoarthritis

**DOI:** 10.1016/j.gendis.2025.101864

**Published:** 2025-09-23

**Authors:** Bo Yang, Feng Li, Jindong Cao, Yuxuan Xie, Yifeng Da, Xuejun Yang, Xiaodong Zhao, Wenhua Xing, Jing Tian

**Affiliations:** a Center for Automated and Innovative Drug Discovery, College of Life Sciences, Northwest University, Xi'an, Shaanxi 710069, China; b Department of Spinal Surgery Center Area A, The Second Affiliated Hospital of Inner Mongolia Medical University, Hohhot, Inner Mongolia 010000, China; c Key Laboratory of Systems Biomedicine (Ministry of Education), Shanghai Center for Systems Biomedicine, Shanghai Jiao Tong University, Shanghai 200240, China; d Key Laboratory of Resource Biology and Biotechnology in Western China, Ministry of Education, School of Medicine, Northwest University, Xi'an, Shaanxi 710069, China

Osteoarthritis (OA), a chronic degenerative joint disorder, is a primary cause of disability, affecting over 520 million people globally.^1^ While age-standardized rates (ASRs) for incidence, prevalence, and disability-adjusted life years (DALYs) are projected to decline slightly annually from 2020 to 2035, absolute case numbers will continue to rise, underscoring OA as a persistent public health challenge.^2^ Therefore, identification of therapeutic targets for OA is of significant clinical importance. The current research on OA drug targets is predominantly confined to blood and single-omics analysis, with a notable absence of integrated, cross-tissue, and multi-omics validation.^3^, ^4^, ^5^ Herein, our objective is to systematically identify tissue-specific priority drug targets for OA through a comprehensive cross-tissue and multi-omics analysis framework.

This study constitutes a secondary analysis of publicly available data. Details of the overall study design and data sources are presented in Figure S1 and Table S1, respectively. Following rigorous screening, we incorporated 2703 cis-eQTLs from 1201 blood druggable genes as instrumental variables (IVs) (Table S2). Through Mendelian randomization (MR) analysis, we identified 16 genes in the blood that were causally associated with 6 OA traits (Fig. 1A and Table S3). Notably, *LYG1* was replicated in the OA GWAS of Guindo-Martínez et al (Fig. S2A and Table S4). Further colocalization analysis revealed that 6 druggable genes (*HLA-DRA*, *NISCH*, *MAST2*, *LYG1*, *IPP*, and *STK25*) in the blood exhibited robust colocalization support (P_H4_ > 0.75) with 4 OA traits (HandOA, KneeHipOA, KneeOA, and TJR) (Fig. 1A and Table S5).

**Figure 1:**
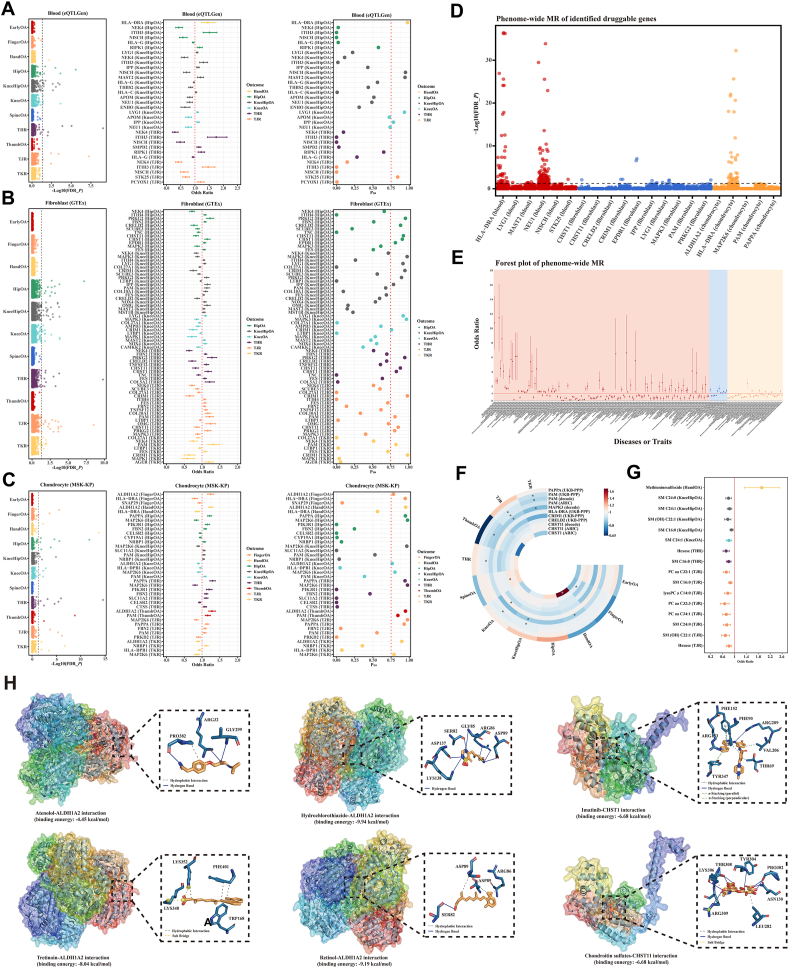
Multi-omics Mendelian randomization combined with single-cell, and spatial transcriptomics: Multidimensional validation of drug targets for osteoarthritis. **(A)** Manhattan plot of OA related druggable genes in blood; Forest plot of OA related druggable genes in blood; Bubble plot of colocalization analysis of OA related druggable genes in blood; **(B)** Manhattan plot of OA related druggable genes in fibroblast; Forest plot of OA related druggable genes in fibroblast; Bubble plot of colocalization analysis of OA related druggable genes in fibroblast; **(C)** Manhattan plot of OA related druggable genes in chondrocyte; Forest plot of OA related druggable genes in chondrocyte; Bubble plot of colocalization analysis of OA related druggable genes in chondrocyte; **(D)** Phenome-wide MR analysis of OA related druggable genes; **(E)** Forest plot between OA related druggable genes and non-OA traits; **(F)** Circular heatmap of druggable genes validated through protein-level MR analysis; **(G)** Forest plot of causal metabolites associated with OA identified in plasma; **(H)** Molecular docking analysis between atenolol and ALDH1A2; Molecular docking analysis between hydrochlorothiazide and ALDH1A2; Molecular docking analysis between tretinoin and ALDH1A2; Molecular docking analysis between retinol and ALDH1A2; Molecular docking analysis between imatinib and CHST1; Molecular docking analysis between chondroitin sulfates and CHST11. The black dashed line depicted in the Manhattan plot signifies the significance threshold, which corresponds to –log10 (0.05). The red line represents an odds ratio of 1. Red symbolizes positive effects, while blue symbolizes negative effects. ∗ denotes statistically significant results, with a corresponding *P* < 0.05.

For fibroblasts, 1308 cis-eQTLs from 1257 genes were selected and used as IVs (Table S6). Subsequently, 32 fibroblast genes were identified as causally associated with the occurrence of 6 OA traits (Fig. 1B and Table S3). *EPDR1* and *LYG1* were replicated (Fig. S2B and Table S4). Additionally, 10 druggable genes (*PRKG2*, *CRELD2*, *CHST11*, *CHST1*, *EPDR1*, *MAPK3*, *LYG1*, *CRIM1*, *IPP*, and *PAM*) in fibroblasts shared genetic variation with 6 OA traits (HipOA, KneeHipOA, KneeOA, THR, TJR, and TKR), demonstrating strong colocalization support (P_H4_ > 0.75) (Fig. 1B and Table S5).

In chondrocytes, 242 cis-eQTLs from 234 genes were selected and utilized as IVs (Table S7), and 16 genes were identified as having causal associations with 9 OA traits (Fig. 1C and Table S3). *MAP2K6* was replicated (Fig. S2C and Table S4). Five druggable genes (*ALDH1A2*, *HLA-DRA*, *PAPPA*, *MAP2K6*, and *PAM*) were detected in chondrocytes, exhibiting strong colocalization support (P_H4_ > 0.75) with 9 OA traits (FingerOA, HandOA, HipOA, KneeHipOA, KneeOA, THR, ThumbOA, TJR, and TKR) (Fig. 1C and Table S5). The above results do not exhibit horizontal pleiotropy and heterogeneity (Tables S3 and S4).

Phenome-wide MR analysis (between 17 druggable genes and 783 non-OA diseases or traits sourced from UKB-SAIGE) revealed that 14 genes (*ALDH1A2*, *CHST1*, *CHST11*, *CRIM1*, *CRELD2*, *IPP*, *LYG1*, *MAPK3*, *MAP2K6*, *NISCH*, *PAM*, *PAPPA*, *PRKG2*, and *STK25*) did not exhibit potential side effects (FDR_*P* < 0.05) (Fig. 1D and E and Table S8).

To further ascertain the genetic association between druggable genes and OA traits, we conducted summary-data-based MR (SMR) and HEterogeneity In Dependent Instruments test (HEIDI) tests on 14 druggable genes and OA traits. The results indicated that 3 genes (*MAST2, LYG1,* and *STK25*) in the blood and 9 genes (*CHST1*, *CHST11*, *CRIM1*, *CRELD2*, *LYG1*, *MAPK3*, *PAM*, *PRKG2*, and *STK25*) in fibroblasts were validated (*P*_SMP < 0.05), with the effect direction aligned with the MR analysis (Fig. S3 and Table S9).

Forty-three cis-pQTLs for 8 proteins (CHST1, CHST11, CRIM1, CRELD2, HLA-DRA, MAPK3, PAPPA, and PAM) were extracted from the ARIC study, UKB-PPP, and deCODE, followed by a protein-level MR analysis (Table S10). CHST1, CHST11, HLA-DRA, MAPK3, and PAM were also validated. The direction of the effect of all positive results (*P* < 0.05) remained consistent with that observed during the druggable genome-wide MR phase (Fig. 1F and Table S11).

To further verify whether OA-related druggable genes increase OA risk through metabolites, we included 154 metabolites and 868 SNPs (Table S12) and conducted metabolic-level MR analysis. Methionine sulfoxide, SM C24:0, SM C24:1, SM (OH) C22:1, SM C16:0, hexose, SM C16:0, PC aa C32:1, lysoPC a C14:0, PC aa C32:3, and PC aa C34:1 was associated with the risk of 5 OA traits (HandOA, KneeHipOA, KneeOA, THR, and TJR) (Fig. 1G and Table S13). Notably, SM C16:0 and PC aa C34:1 are linked to *ALDH1A2* (Table S13). This further confirmed the significant role of *ALDH1A2* in OA risk, potentially regulating the OA process through metabolite production.

Based on single-cell transcriptome data from the GSE176223 dataset, *CHST1*, *PAM*, *HLA-DRA*, *CHST11*, and *PAPPA* were significantly enriched in the synovial tissue of OA patients with pain (*p*_val_adj < 0.05, avg_log2FC > 0.5), whereas *CRELD2*, *PAM*, and *ALDH1A2* were highly expressed in OA patients without pain. Notably, *CHST1* and *PAM* were specifically highly expressed in fibroblasts (Fig. S4 and Table S14), aligning with druggable genome-wide MR results, suggesting a close correlation between their abnormal expression and OA risk. Spatial transcriptome analysis of the GSE253199 dataset revealed that C*RIM1* was significantly enriched (exhibiting a protective effect) in the normal fat pat tissue of 3 sections (*p*_val_adj < 0.05, avg_log2FC > 0.5), whereas *PAM* was up-regulated (indicating a risk-promoting effect) in the pathological tissue of the fourth OA section (*p*_val_adj < 0.05, avg_log2FC > 0.5) (Fig. S5 and Table S15).

The DGIdb database was used to retrieve approved drugs for 14 druggable genes (with no side effects). We identified 4 approved drugs for ALDH1A2, 1 for CHST1, 1 for CHST11, 6 for MAP2K6, and 8 for MAPK3 (Fig. S6 and Table S16). Autodock4 was utilized for further molecular docking to evaluate its therapeutic potential, and the results revealed that all four drugs exhibited strong affinity for ALDH1A2 (binding energy: −4.45 kcal/mol for atenolol, −9.94 kcal/mol for hydrochlorothiazide, −8.04 kcal/mol for tretinoin, and −9.19 kcal/mol for retinol) (Fig. 1H). CHST1 demonstrated strong affinity with imatinib (binding energy: −6.68 kcal/mol) (Fig. 1H). Similarly, CHST11 interacted strongly with chondroitin sulfates (binding energy: −6.68 kcal/mol) (Fig. 1H). These findings further underscore the druggability of ALDH1A2, CHST1, and CHST11, highlighting the immediate translational potential of these targeted drugs.

PPI network analysis utilizing the STRING database identified 14 candidate genes interacting with key molecules in signaling pathways associated with OA. ALDH1A2 interacts with BMP4 of the BMP pathway and CTNNB1 of the WNT pathway through co-expression and text mining, suggesting its role in regulating the BMP/Wnt pathway in OA pathogenesis (Fig. S7). CRIM1 specifically binds to BMP4 and may modulate OA progression via the BMP pathway. MAP2K6 interacts with CTNNB1 (of the WNT pathway) and RELA (of the NF-κB pathway), suggesting its synergistic role across multiple pathways (Fig. S7). MAPK3 interacts with BMP2/4, CTNNB1, NFKB1, VCL, ITGAV, TLN1, NFKB1, REL, RELA, mTOR, and HIF1A, encompassing the BMP, WNT, focal adhesion, NF-κB, mTOR, and HIF pathways, and is associated with known OA targets, such as NGF, TNF, MMP13, IL1B, IL4, IL10, and IL13 (Fig. S7). STK25 is implicated in OA progression through TNF (Fig. S7). This study elucidates the multi-pathway interaction network of candidate targets, providing a mechanistic foundation for their drug development.

In conclusion, this study pioneers the application of a cross-organizational and multi-omics analysis framework to uncover 12 potential therapeutic targets and molecular networks for OA treatment. CHST1, PAM, LYG1, PRKG2, and STK25 emerge as novel targets backed by multi-level evidence. Furthermore, this study underscores the repurposing potential of retinoic acid, retinol, hydrochlorothiazide, atenolol, and imatinib in the treatment of OA.

## CRediT authorship contribution statement

**Bo Yang:** Writing – review & editing, Writing – original draft, Methodology, Investigation, Formal analysis, Data curation. **Feng Li:** Writing – review & editing, Investigation, Funding acquisition, Data curation. **Jindong Cao:** Methodology, Formal analysis. **Yuxuan Xie:** Software, Methodology. **Yifeng Da:** Resources. **Xuejun Yang:** Writing – review & editing. **Xiaodong Zhao:** Writing – review & editing. **Wenhua Xing:** Writing – review & editing, Conceptualization. **Jing Tian:** Writing – review & editing, Writing – original draft, Supervision, Resources, Funding acquisition, Conceptualization.

## Data availability

All data used in this study are publicly available, and all data generated or analyzed during this study are included in this published article and its Supplementary material.

## Funding

This work was funded by the National Natural Science Foundation of China (No. 32170618), the Natural Science Foundation of Inner Mongolia Autonomous Region (China) (No. 2022LHMS08005), and the Fundamental Research Funds for the Central Universities (China) (No. KLSB2023KF-02).

## Conflict of interests

All authors have declared no competing interests.

## Figures and Tables

**figs1:**
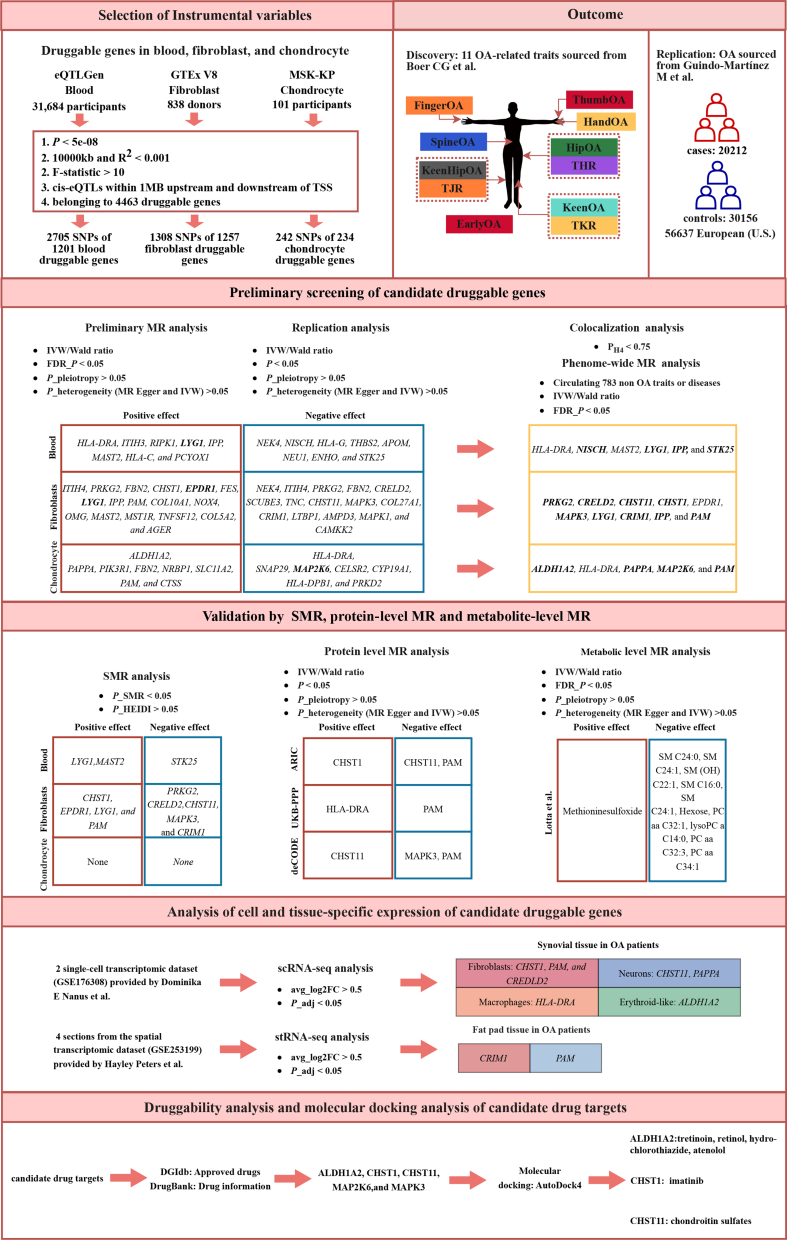


**figs2:**
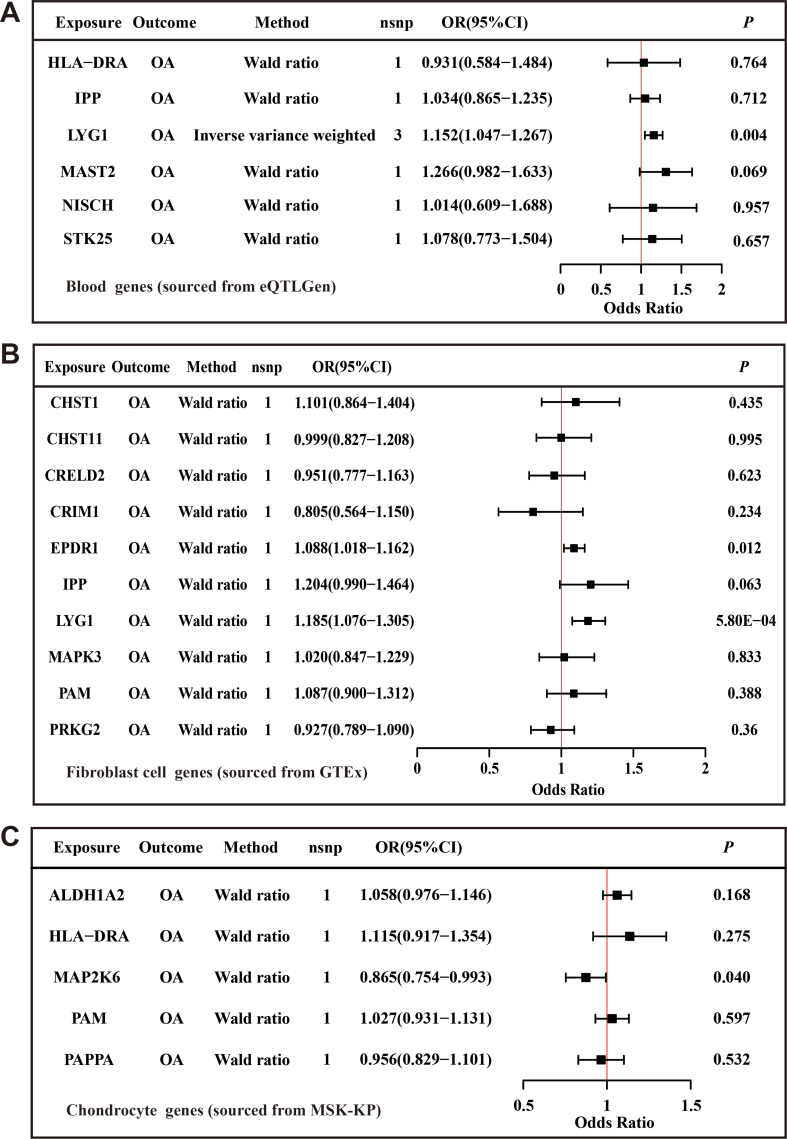


**figs3:**
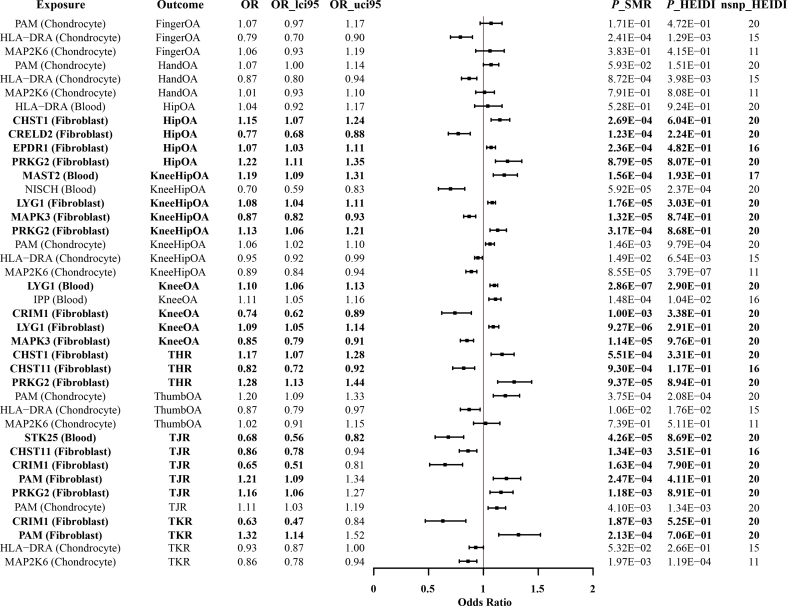


**figs4:**
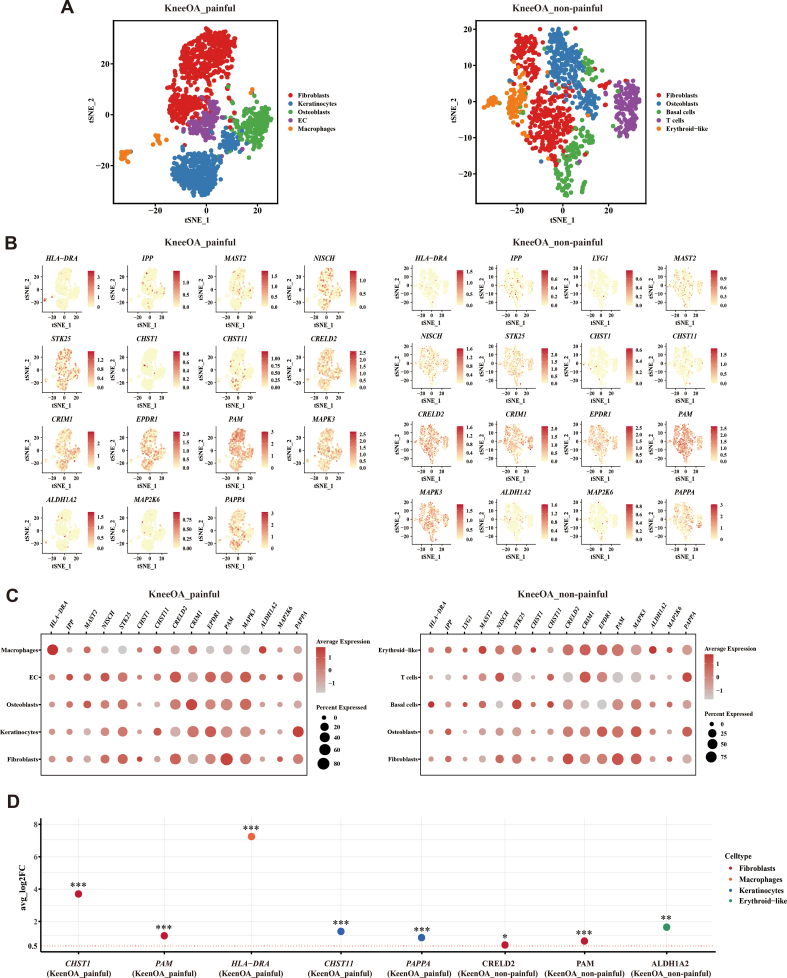


**figs5:**
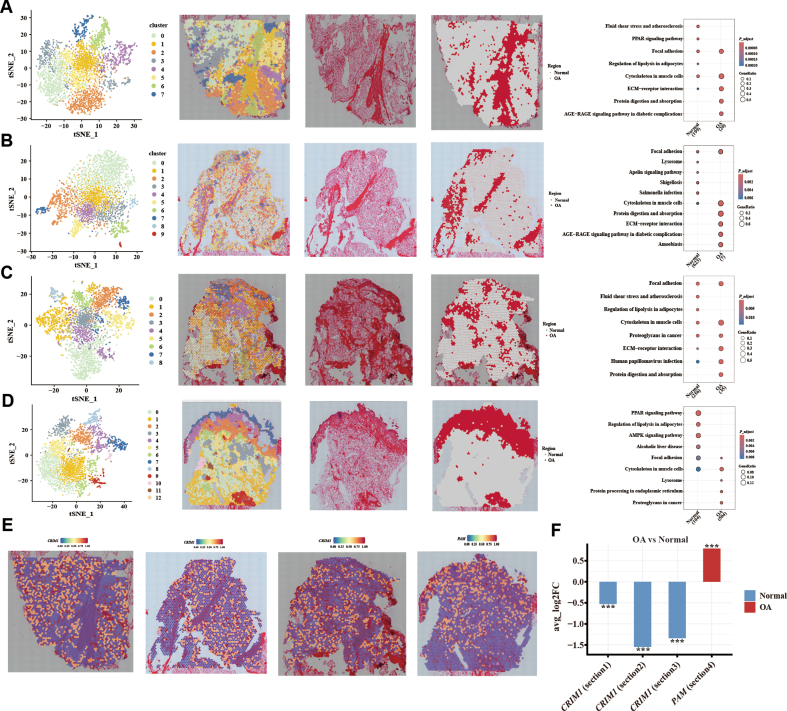


**figs6:**
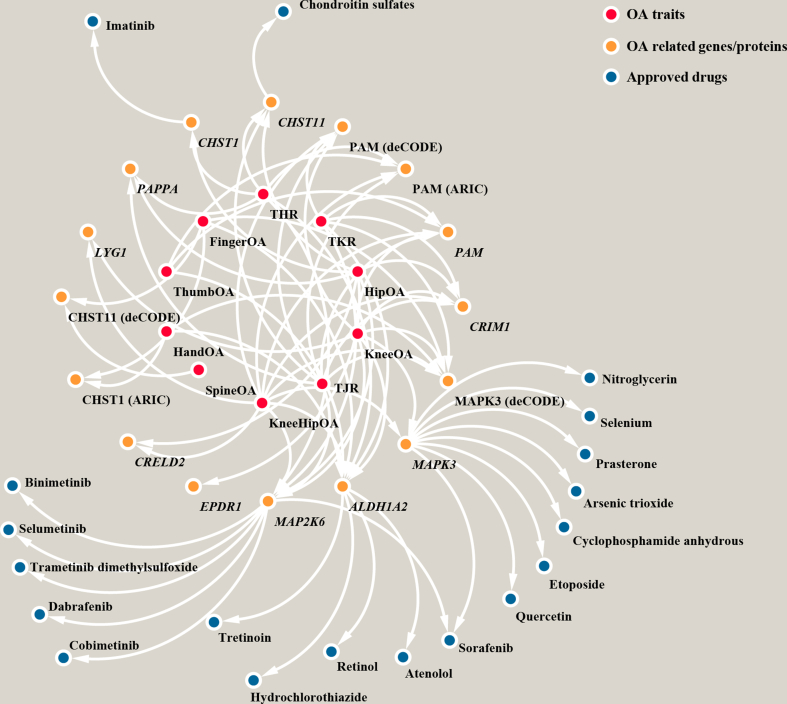


**figs7:**