# Supplementary material for: Multi-omics Mendelian randomization combined with single-cell and spatial transcriptomics: Multidimensional validation of drug targets for osteoarthritis
Source: Genes Dis. 2025 Sep 23;13(5):101864. doi: 10.1016/j.gendis.2025.101864 (PMC13157066; doi:10.1016/j.gendis.2025.101864)
Supplement: Multimedia component 1 [file mmc1.docx]

**Materials & methods**

**Druggable genes list**

The druggable genes discussed in this study were derived from a review by Finan C *et al.* identified 4,479 druggable genes out of 20,300 protein-coding genes. After excluding 16 pseudogenes, the remaining 4,463 genes with unique gene symbols were used in our study ^1^.

**Data source for QTLs**

Blood eQTLs were obtained from eQTLGen consortium, which including 16,989 genes across 31,684 blood samples ^2^. Fibroblast eQTLs were obtained from GTEx V8, which included 20,876 genes from 838 donors ^3^. Chondrocyte eQTLs were derived from the study by Kramer *et al.*, which including 3,782 genes involving 101 individuals ^4^.

The cis-pQTLs of plasma proteins used for protein-level MR validation were sourced from the Atherosclerosis Risk in Communities study (ARIC; including 7,213 European Americans and 4,657 plasma proteins), the UK Biobank Pharma Proteomics Project (UKB-PPP; including 54,219 participants from UKB and 2,923 plasma proteins), and the deCODE database (35,559 Icelandic participants and 4,907 plasma proteins), respectively ^5-7^.

The mGWAS utilized for metabolite-level MR analysis originated from a study conducted by Lotta *et al.*, which assayed 174 metabolites in as many as 86,507 participants ^8^.

**Data source for OA GWAS**

The 11 OA-related traits utilized in this study served as the outcome data for the discovery phase, sourced from the largest OA GWAS meta-analysis dataset of Boer CG *et al.*, encompassing 826,690 participants across 13 databases, with 177,517 individuals diagnosed with OA ^9^. The dataset comprises 11 OA-related traits, including KneeOA (62,497 cases; 333,557 controls), HipOA (36,445 cases; 316,943 controls),KneeHipOA (89,741 cases; 400,604 controls), TKR (18,200 cases; 233,841 controls), THR (23,021 cases; 296,016 controls), TJR (40,887 cases; 327,689 controls), FingerOA (10,804 cases; 255,814 controls), ThumbOA (10,536; 236,919 controls), HandOA (20,901 cases; 282,881 controls), SpineOA (28,372; 305,578 controls), and EarlyOA (6,838 cases; 41,449 controls). The OA GWAS summary statistics from Guindo-Martínez *et al.* (20,212 cases; 30,456 controls) was utilized as a replication dataset ^10^.

**Druggable genome-wide MR analysis**

Druggable genome-wide MR analysis was conducted using the TwoSampleMR package (Version 0.5.7), employing the IVW and wald ratio methods for two-sample MR analysis involving multiple IVs and just one IV, respectively. The IVs included in the druggable genome-wide MR must fulfill the following criteria: (1) cis-eQTLs situated 1 MB upstream and downstream of the transcription start site (TSS); (2) genome-wide significance (*P* < 5 × 10^-8^); (3) independent loci (10000kb, R^2^ < 0.001); and (4) F-statistics >10. False discovery rate (FDR) correction for MR results was performed using the fdrtools (Version 2.7.0) package.

**Colocalization analysis**

The OA targets identified through druggable genome-wide MR analysis were further subjected to colocalization analysis using the coloc (Version 5.2.3) R package, aiming to verify the genetic association between candidate genes and OA traits ^11^. The Bayesian colocalization method calculates the posterior probability based on the following assumptions: (1) H0: no association with either trait in the region; (2) H1: association with trait 1 only; (3) H2: association with trait 2 only; (4) H3: both traits are associated, but have different single causal variants; (5) H4: both traits are associated and share the same single causal variant. If the posterior support probability (P_H4_) of H4 is greater than 0.75, it is considered to have strong colocalization support.

**Phenome-wide MR analysis**

Phenome-wide MR was used to assess the potential side effects and beneficial effects of candidate druggable genes. Phenome-wide MR was conducted based on two-sample MR, with the same IVs included in the analysis as those used in the druggable genome-wide MR. From the SAIGE GWAS, 783 non-OA diseases or traits with cases exceeding 500 were selected as the outcomes for phenome-wide MR analysis ^12^.

**SMR analysis**

To further validate the causal relationship between the candidate druggable genes and OA traits, we utilized the SMR software tool (Version 1.3.1) for in-depth analysis ^13^. In this analysis, we employed cis-eQTLs that were significant (*P* < 5 × 10^-8^) at the genome-wide level.

**Protein-level MR analysis**

To assess the impact of candidate druggable genes on 11 OA traits at the protein level, cis-pQTLs from the ARIC, UKB-PPP, and deCODE databases were utilized as IVs in this study ^5-7^, adhering to the same inclusion criteria as for the druggable genome-wide MR. Subsequently, a two-sample MR analysis was conducted using the IVs of plasma proteins and the GWAS of 11 OA traits. The results were calculated using the IVW and Wald ratio methods. This analytical strategy aimed to uncover the association between candidate genes and OA traits at the protein level.

**Metabolite-level MR analysis**

For MR analysis at the metabolite level, we employed a two-sample MR approach. The utilized IVs originated from the mGWAS conducted by Lotta *et al.* ^8^. Specifically, we selected independent and significant mQTLs satisfying the following criteria: (1) genome-wide significance (*P* < 5 × 10^-8^); (2) independent loci (10000kb, R^2^ < 0.001). To detect statistically significant causal effects, we applied the FDR correction method to adjust for multiple hypothesis testing to identify significant causal effects (FDR_*P* < 0.05). The mapped genes corresponding to the metabolites were obtained through the mGWAS conducted by Lotta *et al.*

**Single cell transcriptome expression analysis**

To identify cell-type-specific expression of candidate druggable genes, we analyzed single-cell RNA-seq data from KneeOA synovial tissue (sourced from Nanus *et al.*) ^14^. Using Seurat (Version 5.2.0), cells with <200 features or <3 genes were excluded. Data were normalized (TPM) and scaled. Cell types were annotated via PanglaoDB ^15^. Differential expression analysis (Wilcoxon test) revealed genes enriched in specific cell types, defined by average log2 fold change (avg_log2FC) > 0.5 and *P* value adjusted for FDR (*p*_val_adj) < 0.05.

**Spatial transcriptome expression analysis**

To delve deeper into the spatial expression patterns of candidate druggable genes within the pathological tissues of patients with OA, this study utilized 4 sections from spatial transcriptomic dataset provided by Hayley Peters *et al.* ^16^. Raw data underwent quality control (cell viability assessment, cycle calibration, filtering) and normalization for variance stabilization using Seurat. PCA dimensionality reduction and Louvain clustering (optimized resolution) identified cell populations, visualized via t-SNE. Fibrosis-high clusters were classified as OA pathological regions, fibrosis-low as normal. Differential expression analysis (*p*_val_adj <0.05, avg_log2FC >0.5) validated region-specific markers. KEGG enrichment analysis confirmed OA-associated pathways in pathological regions, aligning with disease biology. This integrated approach ensured robust identification of spatially resolved gene expression patterns in OA tissues

**Druggability analysis and molecular docking**

To further assess the potential of these candidate druggable genes as drug targets, their druggable profiles were retrieved from the DGIdb database (Version 5.0) ^17^. Autodock4 was utilized for molecular docking analysis with the aim of assessing the affinity between candidate genes and their corresponding approved drugs, thereby facilitating a more comprehensive evaluation of the druggability of candidate genes ^18^. The small-molecule drugs used for docking were sourced from PubChem. The protein structure of ALDH1A2 was retrieved from the PDB database (PDB ID: 4X2Q) ^19^, whereas CHST1 and CHST11 were obtained from the AlphaFold Protein Structure Database ^20^. The interaction mode and binding energy between the proteins and small molecules were visualized using PLIP ^21^.

**PPI analysis**

The STRING (Version 12.0) database was utilized to perform PPI analysis on candidate druggable genes (no side effects), known OA drug targets, and key molecules from OA-related signaling pathways (sourced from Yao *et al.*) ^22^. This analysis aimed to investigate whether the candidate genes could potentially cause OA through interactions with existing drug targets and related molecular pathways.

**References**

1. Finan C, Gaulton A, Kruger FA, Lumbers RT, Shah T, Engmann J, Galver L, Kelley R, Karlsson A, Santos R *et al*. The druggable genome and support for target identification and validation in drug development. *Sci Transl Med* 2017, 9(383).

2. Vosa U, Claringbould A, Westra HJ, Bonder MJ, Deelen P, Zeng B, Kirsten H, Saha A, Kreuzhuber R, Yazar S *et al*. Large-scale cis- and trans-eQTL analyses identify thousands of genetic loci and polygenic scores that regulate blood gene expression. *Nat Genet* 2021, 53(9):1300-1310.

3. Consortium GT. The GTEx Consortium atlas of genetic regulatory effects across human tissues. *Science* 2020, 369(6509):1318-1330.

4. Kramer NE, Byun S, Coryell P, D'Costa S, Thulson E, Kim H, Parkus SM, Bond ML, Klein ER, Shine J *et al*. Response eQTLs, chromatin accessibility, and 3D chromatin structure in chondrocytes provide mechanistic insight into osteoarthritis risk. *Cell Genom* 2025, 5(1):100738.

5. Rooney MR, Chen J, Echouffo-Tcheugui JB, Walker KA, Schlosser P, Surapaneni A, Tang O, Chen J, Ballantyne CM, Boerwinkle E *et al*. Proteomic Predictors of Incident Diabetes: Results From the Atherosclerosis Risk in Communities (ARIC) Study. *Diabetes Care* 2023, 46(4):733-741.

6. Sun BB, Chiou J, Traylor M, Benner C, Hsu YH, Richardson TG, Surendran P, Mahajan A, Robins C, Vasquez-Grinnell SG *et al*. Plasma proteomic associations with genetics and health in the UK Biobank. *Nature* 2023, 622(7982):329-338.

7. Ferkingstad E, Sulem P, Atlason BA, Sveinbjornsson G, Magnusson MI, Styrmisdottir EL, Gunnarsdottir K *et al*. Large-scale integration of the plasma proteome with genetics and disease. Nat Genet. 2021 Dec;53(12):1712-1721.

8. Lotta LA, Pietzner M, Stewart ID, Wittemans LBL, Li C, Bonelli R, Raffler J, Biggs EK, Oliver-Williams C, Auyeung VPW *et al*. A cross-platform approach identifies genetic regulators of human metabolism and health. *Nat Genet* 2021, 53(1):54-64.

9. Boer CG, Hatzikotoulas K, Southam L, Stefansdottir L, Zhang Y, Coutinho de Almeida R, Wu TT, Zheng J, Hartley A, Teder-Laving M *et al*. Deciphering osteoarthritis genetics across 826,690 individuals from 9 populations. *Cell* 2021, 184(18):4784-4818 e4717.

10. Guindo-Martinez M, Amela R, Bonas-Guarch S, Puiggros M, Salvoro C, Miguel-Escalada I, Carey CE, Cole JB, Rueger S, Atkinson E *et al*. The impact of non-additive genetic associations on age-related complex diseases. *Nat Commun* 2021, 12(1):2436.

11. Giambartolomei C, Vukcevic D, Schadt EE, Franke L, Hingorani AD, Wallace C, Plagnol V. Bayesian test for colocalisation between pairs of genetic association studies using summary statistics. PLoS Genet. 2014 May 15;10(5):e1004383.

12. Bi W, Zhou W, Zhang P, Sun Y, Yue W, Lee S. Scalable mixed model methods for set-based association studies on large-scale categorical data analysis and its application to exome-sequencing data in UK Biobank. *Am J Hum Genet* 2023, 110(5):762-773.

13. Zhu Z, Zhang F, Hu H, Bakshi A, Robinson MR, Powell JE, Montgomery GW, Goddard ME, Wray NR, Visscher PM *et al*. Integration of summary data from GWAS and eQTL studies predicts complex trait gene targets. *Nat Genet* 2016, 48(5):481-487.

14. Nanus DE, Badoume A, Wijesinghe SN, Halsey AM, Hurley P, Ahmed Z, Botchu R, Davis ET, Lindsay MA, Jones SW. Synovial tissue from sites of joint pain in knee osteoarthritis patients exhibits a differential phenotype with distinct fibroblast subsets. *EBioMedicine* 2021, 72:103618.

15. Franzen O, Gan LM, Bjorkegren JLM. PanglaoDB: a web server for exploration of mouse and human single-cell RNA sequencing data. *Database (Oxford)* 2019, 2019.

16. Peters H, Potla P, Rockel JS, Tockovska T, Pastrello C, Jurisica I, Delos Santos K, Vohra S, Fine N, Lively S *et al*. Cell and transcriptomic diversity of infrapatellar fat pad during knee osteoarthritis. *Annals of the Rheumatic Diseases* 2025, 84(2):351-367.

17. Cannon M, Stevenson J, Stahl K, Basu R, Coffman A, Kiwala S, McMichael JF, Kuzma K, Morrissey D, Cotto K *et al*. DGIdb 5.0: rebuilding the drug-gene interaction database for precision medicine and drug discovery platforms. *Nucleic Acids Res* 2024, 52(D1):D1227-D1235.

18. Morris GM, Huey R, Lindstrom W, Sanner MF, Belew RK, Goodsell DS, Olson AJ. AutoDock4 and AutoDockTools4: Automated docking with selective receptor flexibility. *J Comput Chem* 2009, 30(16):2785-2791.

19. Berman HM, Westbrook J, Feng Z, Gilliland G, Bhat TN, Weissig H, Shindyalov IN, Bourne PE. The Protein Data Bank. *Nucleic Acids Res* 2000, 28(1):235-242.

20. Jumper J, Evans R, Pritzel A, Green T, Figurnov M, Ronneberger O, Tunyasuvunakool K, Bates R, Zidek A, Potapenko A *et al*. Highly accurate protein structure prediction with AlphaFold. *Nature* 2021, 596(7873):583-589.

21. Adasme MF, Linnemann KL, Bolz SN, Kaiser F, Salentin S, Haupt VJ, Schroeder M. PLIP 2021: expanding the scope of the protein-ligand interaction profiler to DNA and RNA. *Nucleic Acids Res* 2021, 49(W1):W530-W534.

22. Yao Q, Wu X, Tao C, Gong W, Chen M, Qu M, Zhong Y, He T, Chen S, Xiao G. Osteoarthritis: pathogenic signaling pathways and therapeutic targets. *Signal Transduct Target Ther* 2023, 8(1):56.

**Fig. S1** **Flowchart of the study design**

OA, Osteoarthritis; MR, Mendelian Randomization; SMR, Summary-data-based Mendelian Randomization.

**Fig. S2 Replication of candidate druggable genes**

**(A)** Replication of candidate druggable genes in blood; **(B)** Replication of candidate druggable genes in fibroblast; **(C)** Replication of candidate druggable genes in chondrocyte.

**Fig. S3 Validation of candidate druggable genes via SMR**

Bold font indicates the positive results of SMR analysis (*P*_SMR < 0.05 and *P*_HEIDI < 0.05)

**Fig. S4 Cell-specific expression patterns of candidate druggable genes in synovial tissue of patients with OA**

**(A)** Classification of cellular subpopulations using t-SNE method; **(B-C)** Expression patterns of candidate druggable genes; **(D)** The cell types exhibiting specific expression of candidate druggable genes. The red dashed line signifies the average_log2FC, with a corresponding value of 0.5. **p*_val_adj < 0.05, ***p*_val_adj < 0.01, ****p*_val_adj < 0.001.

**Fig. S5 Spatially-specific expression of candidate druggable genes in the fat pad tissues of patients with OA**

**(A-D)** Classification of pathological tissues and normal tissues; **(E)** Spatial expression of CRIM1 and PAM within tissue sections; **(F)** Significant differential expression of CRIM1 and PAM. **p*_val_adj < 0.05, ***p*_val_adj < 0.01, ****p*_val_adj < 0.001.

**Fig. S6 OA trait-candidate druggable gene/protein-targeted drug network**

**Fig. S7 PPI analysis between candidate druggable genes and known targets of OA as well as key molecules involved in OA-related signaling pathways**
